# Hot Spots for Protein Partnerships at the Surface of Cholinesterases and Related α/β Hydrolase Fold Proteins or Domains—A Structural Perspective

**DOI:** 10.3390/molecules23010035

**Published:** 2017-12-23

**Authors:** Yves Bourne, Pascale Marchot

**Affiliations:** Centre National de la Recherche Scientifique, Aix-Marseille Université, “Architecture et Fonction des Macromolécules Biologiques” Laboratory, 13288 Marseille, France

**Keywords:** α/β hydrolase fold, binding surface, cholinesterase-like domain, complex interface, crystal structure, four-helix bundle, dimer, tetramer, functional partnership

## Abstract

The hydrolytic enzymes acetyl- and butyryl-cholinesterase, the cell adhesion molecules neuroligins, and the hormonogenic macromolecule thyroglobulin are a few of the many members of the α/β hydrolase fold superfamily of proteins. Despite their distinctive functions, their canonical subunits, with a molecular surface area of ~20,000 Å^2^, they share binding patches and determinants for forming homodimers and for accommodating structural subunits or protein partners. Several of these surface regions of high functional relevance have been mapped through structural or mutational studies, while others have been proposed based on biochemical data or molecular docking studies. Here, we review these binding interfaces and emphasize their specificity versus potentially multifunctional character.

## 1. Introduction

Acetylcholinesterase (AChE) is the enzyme responsible for the rapid hydrolysis of the neurotransmitter ACh in the cleft of cholinergic synapses in the peripheral and central nervous systems (PNS, CNS) [1,2,3]. Due to this prominent role in neurotransmission, AChE is the target of a variety of reversible or irreversible inhibitors, ranging from natural or synthetic organic compounds such as insecticides and organophosphorus (OP) nerve agents, to natural or engineered peptidic inhibitor(s) such as animal toxins and antibodies, and to the first generation of anti-Alzheimer symptomatic drugs [4].

AChE is the lead member of the α/β-hydrolase fold superfamily of proteins, which encompasses structurally related proteins with diverse catalytic and non-catalytic functions [5,6,7]. While the catalytic members of the superfamily are dominated by classical hydrolases, including the AChE cousin, butyrylcholinesterase (BChE), which is present in high amounts in liver and plasma but whose role in mammalian physiology is unclear; the non-catalytic members, which are devoid of a functional catalytic triad, include the ectodomain of the cell adhesion molecules neuroligin (NLG) in many species and glutactin, gliotactin, neurotactin in insects [8]; and the C-terminal, intramolecular chaperone domain of the hormone precursor thyroglobulin (TG) in vertebrates [9].

The pioneering crystal structure of *Torpedo californica* AChE (TcAChE) revealed the atypical location of the Glu/His/Ser catalytic triad, which is buried nearly centro-symmetric to the subunit at the bottom of a long and narrow gorge but still, binds reversible and irreversible organic competitive inhibitors of natural or synthetic origins [10]. At the surface of the subunit and rim of the gorge, the peripheral anionic site (PAS) encompasses overlapping binding loci for a variety of positively charged, reversible non-competitive modulators of catalysis [11,12], such as the natural peptidic toxin from snake venom, fasciculin-2 (Fas2), or the engineered inhibitory antibodies Elec403 and Elec410, or the small organic compounds propidium and gallamine. The unique topology of the AChE active center gorge with its two remote binding subsites has favoured the design of bifunctional inhibitors spanning the full length of the gorge and displaying enhanced potency and selectivity, compared to their single-site precursors [13,14].

In addition to its “classical” role in terminating synaptic transmission, AChE is proposed to play non-classical roles including cell adhesion, neurite outgrowth through recognition of laminin [15] and potentiation of amyloid-β peptide nucleation into pathogenic fibrils [16] (for a review see [3]). Experimental evidences obtained in vitro or *in cellula* for involvement of the PAS along with computational studies have been reported, but no experimental structural data are available (for a review see [17] in this issue).

Alternative splicing of the C-terminal exon, post-translational modifications and association of the AChE subunit with proline-rich peptides generate an array of tissue-specific quaternary molecular forms that are either soluble or anchored in membranes or extracellular matrices in the PNS and CNS [1,18]. In the CNS, the C-terminal amphipathic T peptide, also defined as the tryptophan amphiphilic tetramerization (WAT) domain, interacts with the hydrophobic proline-rich attachment domain (PRAD) of the non-catalytic proline-rich membrane-anchoring (PRiMA) protein to form tetramers, while the alternative C-terminal H peptide produces GPI-anchored dimers [1,19,20,21]. At the neuromuscular junction, AChE forms asymmetric oligomers consisting of one to three tetramers of subunits whose WAT domains are attached to the PRAD of a non-catalytic collagen-like Q (ColQ) subunit linked to the basal lamina [1,19,22,23]. Similarly, the BChE catalytic subunits form tetramers non-covalently attached to a polyproline-rich peptide from lamellipodin [24], as exemplified elsewhere in this issue for the rare C5 genetic variant of BChE [25]. 

In addition to the ligand-subunit and subunit-subunit interfaces evidenced in crystal structures of cholinesterases (ChE) and their complexes, other functional interfaces for ligand binding onto the α/β-hydrolase fold domain were identified in structures of neuroligins bound with their neurexin [26,27,28] and MDGA partners [29,30,31,32]. Here again, alternative protein partners of the neuroligins have been reported, such as PTPRT, TSP-1, GluN1 (reviewed in [33]), punctin/MADD-4 [34,35] or Slitrk3 [36], yet structural information on these complexes is not available.

Here, we briefly review the main findings from structural studies documenting the mode of binding of peptidic partners to the α/β-hydrolase fold domain of AChE and ChE-like proteins. This compilation emphasizes the diversity of the structural to functional adaptations of the α/β-hydrolase fold, highlights the multifunctional property of some of the binding interfaces, and provides clues toward investigating possible molecular determinants associated with non-classical functions of AChE.

## 2. Results

### 2.1. The Dimer and Tetramer Interfaces

**Figure 1:**
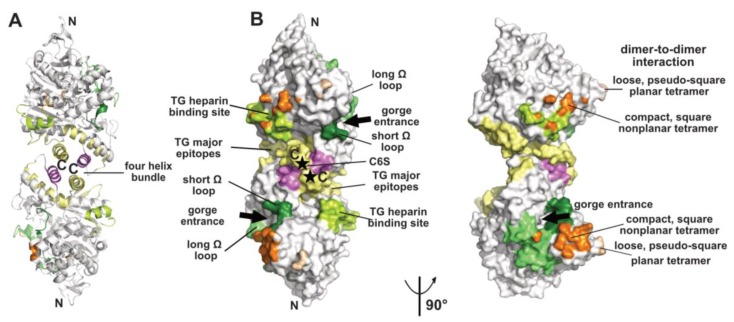
Overview of a representative α/β-hydrolase fold molecule (mAChE, PDB code 1J06) displayed as a dimer of subunits related by a two-fold symmetry axis, in ribbon (**A**) and surface modes (**B**) (with the left and right dimers oriented 90° from each other). The functional surfaces that mediate peptidic ligand binding and tetrameric assembly are color-coded differentially and labeled: helices α3(7,8) and α10 that form the four-helix bundle at the dimer interface are displayed in yellow and violet and the long and short Ω loops that form part of the PAS are in medium green and dark green, respectively. The major epitopes (of which one corresponds to helix α10) and the heparin-binding site in hTG are shown in yellow and light green, respectively, while the attachment sites for chondroitin 6-sulfate oligosaccharide moiety at the C-termini of the subunits are indicated by asterisks and labeled C6S. The surfaces buried at the EeAChE dimer-to-dimer interfaces are shown in wheat for the loose, pseudo-square planar tetramer (modeled from PDB code 1C2B) and in orange for the compact, square nonplanar tetramer (modeled from PDB code 1C2O). The N- and C-termini of each subunit in the dimer and the two active-center gorge entrances are indicated. The nomenclature used for the secondary structure elements is that of [37].

The crystallized covalent dimer of TcAChE was found to assemble through a tightly packed parallel four-helix bundle involving helix α3(7,8) and the C-terminal helix α10 from each subunit [10]; nomenclature from [37]). As a result, the C-terminal ends of the two subunits converge towards the same direction, while the active center gorge entrances lie on opposite faces of the dimer. Recombinant soluble AChE from mouse (mAChE) and natural AChE from snake venom (BfAChE), both devoid of the C-terminal amphipathic helix and intersubunit disulfide-linking cysteines, behave as monomers in dilute solution, yet in concentrated solutions and in the crystals they form the same homodimer as the *Torpedo* enzyme [38,39,40]. The dimer interface, which buries a surface area of ~850–900 Å^2^ (~5% of the molecular surface of the AChE subunit) (Figure 1), is dominated by hydrophobic interactions, two features arguing that the Cys-containing C-terminal segment is not a major determinant for dimer formation. This homodimeric assembly, consistently observed amongst AChE species, delineates the starting point for the formation of tetramers (see below), as well as more complex structures as exemplified by the asymmetric trimer of tetramers coordinated by a ColQ subunit found at the neuromuscular junction [41].

Along the structural history of the homologous BChE, a more complex situation was encountered. Recombinant human BChE (hBChE) expressed as a fully glycosylated monomer from insect cells was found to form the canonical dimer [42], as does a non-glycosylated variant expressed from bacteria (see [43] in this issue). In contrast, engineered monomers with a reduced number of N-glycans and with either truncated or intact C-termini, expressed from CHO cells, formed a “twisted” dimer with a non-parallel, ~45° rotated bundle (buried surface area: ~360 Å^2^) and where the two gorge entrances are located on the same side of the dimer [44,45], or a totally different assembly with divergent C-termini and no four-helix bundle (buried surface area: ~480 Å^2^) [46], respectively. The twisted dimer, presumed to be generated by the crystal packing, may also reflect an alternate, minor abundance conformation of the BChE dimer in solution. The mechanistic or evolutionary basis of the non-canonical dimer is unknown, yet it raises the question of which two molecules, among those present in the crystal unit cell, are more likely to reflect a dimer (see below).

The ectodomain of the non-catalytic cell adhesion molecule, neuroligin, shares ~35% sequence identity with that of AChE. This ectodomain, which is devoid of a C-terminal intersubunit disulfide-linking cysteine and is expressed as a monomer, also generates canonical dimers in concentrated solution and in the crystal [26,27,47]. In addition to dimer formation, the structures of neuroligins NLG1, NLG2 and NLG4 in absence or presence of a bound neurexin partner also provide valuable information on the molecular similarities versus particularities of the neuroligins compared with AChE (see below). A natural Arg to Cys residue substitution within the four-helix bundle of BChE and neuroligin NLG3 associated with human congenital diseases have been shown to impair proper ER trafficking through disruption of the canonical dimer of subunits (for a review see [48]). Whether the ectodomains of the insect cell adhesion molecules, neurotactin and glutactin, also form concentration-driven dimers is not documented, yet analysis of their amino acid sequences suggests that their α3(7,8) and α10 helices contain suitable determinants for dimer formation.

Finally, the ChE-like domain of TG triggers functional homodimerization of the whole macromolecule via non-covalent interactions thought to involve a four-helix bundle as found in the ChEs and neuroligins (for a review see [49]). This event is required for proper ER trafficking to the thyrocyte and storage of TG. In human TG, the major epitopes for autoimmune thyroid disease-related antibodies involve the surface loop between helices α3(7,8) and α4(7,8), the C-terminal helix α10 and strands β9 and β10, all being located in the C-terminal part of the subunit, suggesting that antibody binding could destabilize or disrupt the dimer [50,51] (Figure 1). In contrast, presence of a bulky chondroitin 6-sulfate oligosaccharide moiety linked downstream to helix α10, and reported to enhance hormone formation and limit proteolytic accessibility of the C-terminus [52], may contribute to dimer stability (Figure 1). 

Functional forms of AChE and BChE also comprise tetramers of catalytic subunits coordinated by a PRAD or PRiMA peptide [1,19,22,23]. The quaternary organisation of natural AChE tetramers, isolated from the ColQ-coordinated dodecamers found in the gymnotus electroplax and similar to the form found at the neuromuscular junction, was approached at low-resolution by crystallography [53,54]. Two related assemblies of dimers of canonical dimers were observed, suggesting conformational flexibility of the oligomer: a loose, pseudo-square planar tetramer with four accessible gorge entrances, and a compact, square non-planar tetramer with two accessible gorge entrances only, but none of the C-terminal WAT domains nor the coordinating PRAD peptides were resolved (Figure 1). For the loose tetramer, the limited contacts found at the dimer-to-dimer interfaces and the large empty space in the center raised a question as to how they could be tethered together, and suggested conformational disorder (rather than absence in the crystals) of a centrally positioned tetramerisation domain. For the compact tetramer, the wider dimer–to-dimer interfacial area (~330 Å^2^) and the possible projection of a deported (and disordered as well) tetramerization domain out of the tetramer main core offer a plausible interpretation. An antiparallel architecture of the tetramerization domain was subsequently approached through the superhelical structure of an antiparallel PRAD-WAT complex made from synthetic peptides in a 1:4 molar ratio [55]. Together, these data provided complementary bases for exploration of the tetramer coordination by molecular modeling and dynamics simulations [56,57] (for a review see [17] in this issue). Other tetrameric assemblies were observed in crystal structures solved from recombinant monomers of AChE, which however formed canonical dimers ([58] and further analyzed in [56]), or of BChE, which formed non-canonical dimers [46] (see above), but experimental in vitro data that would support them physiologically are not available.

### 2.2. The Peripheral Anionic Site

**Figure 2:**
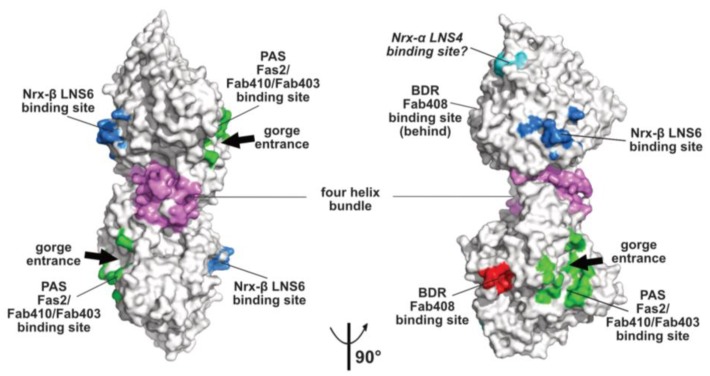
Overall views, oriented 90° from each other, of the mAChE dimer in surface mode with the binding surfaces for the peptidic ligands Fas2 (PDB code, 1KU6), Fab410 (modeled from PDB code 4QWW) and Fab403 at the AChE PAS displayed in green, those for Fab408 in the back door region of AChE in red, those for neurexin-β or the neurexin-α LNS6 domain on neuroligins NLG1 and NLG4 in blue (modeled from PDB code 2XB6), and those for the neurexin-α LNS4 domain on neuroligins in cyan. Helices α3(7,8) and α10 in the dimer four-helix bundle are displayed in violet.

The topology of the PAS at the rim of the AChE active center gorge and the major contribution of residue Trp279 to its functionality were first illustrated by the crystal structure of TcAChE in complex with the bis-quaternary inhibitor, decamethonium [59], and then further emphasized by structures of mAChE complexes with the phenylphenanthridinium inhibitors, decidium and propidium, and the pyrogallol inhibitor, gallamine [60]. The PAS is also a binding site for the reversible inhibitors, BW284C51 and d-tubocurarine [61]. A more complete description of the PAS extended surface arose from structures of mAChE and TcAChE bound with the 7kDa peptidic toxin, Fas2, from snake venom, in which bound Fas2 at the gorge entrance sterically restrains ligand access to the active center [38,62]. Three anchoring points for Fas2 at the PAS involve the so-called long and short Ω loops and the α(1)7,8-α(2)7,8 loop, and contribute an interfacial surface area of ~1040 Å2 (~5% of the molecular surface of the AChE subunit) (Figure 2). This surface area, which is reflected in the subnanomolar to picomolar affinities (Kd and Ki values) of Fas2 for the sensitive AChE species, covers most of the narrower binding loci for the non-competitive, lower-affinity organic AChE inhibitors above cited.

Canonical AChE dimers symmetrically bind two Fas2 molecules, as does each of the two dimers in the loose EeAChE tetramer [53,54]. In contrast, the compact AChE tetramer, in which two of the four PAS are buried at the dimer-to-dimer interface, would not be expected to bind more than two Fas2 molecules. Yet, no evidence for a partially only occupied tetramer at saturating Fas2 concentration in solution could be obtained, a feature supporting existence of several conformational states of the tetramer in solution and their differential trapping in the crystals [54].

Monoclonal antibodies Elec403 and Elec410, which were raised against EeAChE and inhibit only EeAChE, and both EeAChE and BfAChE, respectively, were found to inhibit EeAChE in a mutually exclusive manner and competitively with the PAS ligands Fas2 and propidium [63,64]. The crystal structure of BfAChE bound with the Elec410 fragment, Fab410, shows the Fab410 molecule sited on the long Ω loop on one side of the gorge rim where it partially occludes the gorge entrance, a position consistent with the residual activity of the complex (~7% of that of unliganded EeAChE) [40]. Here, Fab410 buries a surface area of ~900 Å^2^ (~5% of the molecular surface of the AChE subunit), corresponding to a 40% overlap with the Fas2 binding interface (Figure 2). Despite the limited overlap of the backbones of bound Fab410 and Fas2, the spatial arrangements of key positively charged and aromatic side chains from the interacting Fab410 CDRs and Fas2 loops surrounding the gorge rim are remarkably conserved, an observation consistent with the cationic nature of the two inhibitors [40,65].

Binding of Elec403 and its Fab403 congener at the EeAChE PAS inhibit the catalytic activity almost completely (residual activity of the complex: ~1% of that of the unliganded enzyme) suggesting complete occlusion of the gorge entrance, as seen with Fas2 [64]. No crystal structure of a Fab403-AChE complex is available, but the comprehensive mutational approach conducted to map the binding sites of the “Elec” antibodies led to circumscribe a surface area of ~900 Å^2^ (~5% of the molecular surface of the AChE subunit) and predict 60% and 100% overlaps with the Fab410 and Fas2 binding interfaces (Figure 2).

The “non classical” functions of AChE involve associations with peptidic ligands of the PAS, as shown by competition experiments with standard PAS inhibitors (for a review see [3]). These ligands include amyloid-β peptides ([16], and references therein); [58] and the β-chain of laminin-1 ([15], and references therein). Amyloid-β binding does not inhibit the enzyme activity, suggesting that the reported competition with bound PAS inhibitors may involve steric proximity to the gorge rim rather than overlapping binding at the gorge entrance. Laminin binding was suggested to involve the laminin-binding Leu-Arg-Glu motif present in the PAS region in *Torpedo* species AChE [66], but no information about the influence of laminin binding on the enzyme activity is available. For both ligands, several binding sites at the AChE surface in addition to the PAS were predicted by theoretical docking studies [67,68].

The PAS of AChE may also be the site of association of surface loops from adjacent subunits, as observed in the crystal state for the compact conformation of the EeAChE tetramer (Figure 1, see above) and for the tetrameric assembly of mAChE monomers [58]. In fact, structural superimposition of the amyloid-β peptide 1-40 with that of the short Ω loop as bound to the mAChE PAS pointed to positional alignment of several identical or similar side chains [58]. However, evidence for occurrence of such interactions in physiological solution conditions is missing.

There is no equivalent of a PAS at the entrance of the BChE gorge, essentially due to absence of the key aromatic residues that dictate the AChE PAS functionality, as illustrated by a comparison of the binding mode of reversible inhibitors of AChE and BChE (see [69] in this issue). In fact, the physiological role of this enzyme secreted from the liver into the plasma has long been elusive, besides serving as a bio-scavenger protecting AChE from inhibition by circulating organophosphorous toxicants [70]. Recently however, an unexpected role was reported, of deacylating hydrolysis of the acylated peptide, ghrelin, which stimulates hunger and food-seeking (for a review see [71]). Whether the active center or a surface site of BChE is involved in this reaction is unknown, but the ghrelin size (3.2 kDa) may preclude its access to the bottom of the active center gorge. Incidentally, sequence comparison points to intriguing similarity of the positively charged loop II of Fas2 and a very cationic peptide of ghrelin. 

There is no active center gorge nor PAS in and on the neuroligin subunit, where this region is substantially reshaped through significant rearrangement of several surface loops compared with AChE (including the Cys-loop that corresponds to the AChE long Ω loop), whereas loops located at the opposite face of the subunit and involved in neurexin binding are much conserved, an observation suggesting the presence of binding sites for alternative neuroligin partners on the “PAS side” of the subunit [27,72] (see below).

### 2.3. The Neurexin Binding Sites

**Figure 3:**
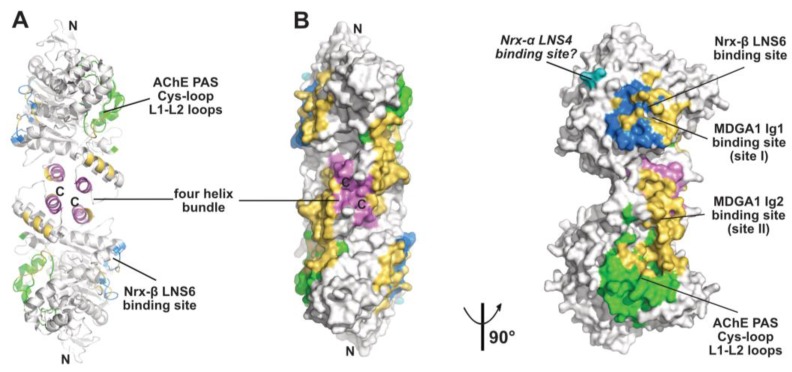
Overall view of the neuroligin NL2 dimer displayed in the ribbon (**A**) and surface modes (**B**) (with the left and right NL2 dimers oriented 90° from each other), with the binding surfaces for neurexin-β or the neurexin-α LNS6 domain (modeled from PDB code 5XEQ) and for the neurexin-α LNS4 domain displayed in blue and cyan, respectively, and those for the MDGA1 Ig1 and Ig2 domains in gold. The Cys-loop and L1-L2 loops, which correspond to the long Ω loop and vicinal loops at the AChE PAS, are displayed in green and helices α3(7,8) and α10 in the dimer four-helix bundle in violet. The N- and C-termini are indicated.

The α/β-hydrolase fold ectodomain of the postsynaptic cell adhesion molecule neuroligin associates trans-synaptically with the ectodomains of the presynaptic long α- and short β-neurexins. Neurexins α encompass six laminin-neurexin-sex hormone binding-protein (LNS) domains intercalated by three epidermal growth factor (EGF)-like domains while neurexins β contain a single LNS domain that is identical to the neurexin-α LNS6 domain. Crystal structures of neuroligins NLG1 and NLG4 bound with neurexin-β provide valuable information on the molecular determinants involved in complex formation [26,27,28,73,74]. Neurexin-β binds on a relatively flat surface of area ~600 Å^2^ (~3% of the molecular surface of the neuroligin subunit) that principally involves the α(4)6,7-β7 loop with minor contribution from the α(1)7,8-α8 and α5,6-β6 loops (Figure 2 and Figure 3). Compared with the Fas2 binding site on AChE (see above), the neurexin-β binding site on neuroligin is located on the opposite side of the α/β-hydrolase fold subunit. Comparison of neurexin-bound versus -unbound neuroligin structures shows that neurexin-β binding to NLG1 does not involve any significant conformational change in their respective binding surfaces, while its binding to NLG4 is accompanied by concerted positional rearrangement of several side chains at the NLG4 binding surface [72]. Strikingly, exploration of the NLG4 subunit on the face opposite to the neurexin-β binding site, i.e., the face corresponding to the PAS and Fas2 binding site on AChE, also revealed concerted conformational rearrangements of several surface loops, including the Cys-loop that corresponds to the Ω loop in AChE, despite their remote location relative to bound neurexin. Although no neuroligin partner distinct from the neurexins was documented at that time, this binding site was hypothesized to be involved into recognition and binding of a “still non-identified second neuroligin partner” [72]. Since then, alternative neuroligin partners have been described (see below).

No structure of a neuroligin in complex with a long neurexin-α is available, yet the molecular conformation and local flexibility of the elongated neurexin-α ectodomain were well documented through analysis of constructs comprising the LNS5-6, LNS2-6 and LNS1-6 domains and their intervening EGF domains [75,76,77]. (In fact, due to inherent flexibility of the LNS1 domain and resulting lack of visualization, the structures of the latter two constructs were found identical.) Structural overlay of neurexin-α LNS2-6 onto neurexin-β (equivalent to LNS6, see above) bound to neuroligin NLG1 or NLG4 positioned the LNS4 domain proximal to a flexible surface loop at the neuroligin surface, corresponding to the β2-β3 loop in AChE and located in the N-terminal part of the α/β-hydrolase fold subunit (Figure 2 and Figure 3). This suggests existence of a secondary interaction site for the long neurexin-α at the neuroligin surface, with both sites being located on the same subunit [76,77]. 

In the ChE-like domain of human and rat TG, the α4(6,7)-β7 surface region homologous to the neurexin-β binding site on neuroligin contains a consensus sequence for heparin binding that may mediate binding of megalin (or gp330), a huge membrane-tethered glycoprotein receptor whose multimodular ectodomain participates to the endocytosis process required for thyroid hormone release [78,79,80] (Figure 1). In crystalline BChE, a dense network of polar interactions involves residues located within this same region to coordinate symmetry-related molecules, a feature that could also reflect a still non-identified functionality [42].

### 2.4. The MDGA Binding Interfaces

The meprin, A5 protein, and receptor protein-tyrosine phosphatase mu [MAM] domain-containing GPI-anchored (MDGA) proteins come as two major, highly similar isoforms whose ectodomains comprise (from the N- to C-terminal) six immunoglobulin (Ig)-like domains followed by one fibronectin type III (FN-III) domain and one MAM domain. *In cellula*, MDGA1 was reported to bind neuroligin NLG2 competitively with neurexin binding, while MDGA2 would bind both neuroligins NLG1 and NLG2 (reviewed in [32]). Structural exploration of this novel partnership of the neuroligin α/β-hydrolase fold ectodomain and its functional implications reveled an unexpected concerted contribution of both subunits in the NLG2 dimer for binding of the long MDGA ectodomain [29,30,31]. Indeed, in the complexes, the MDGA1 Ig1 and Ig2 domains, which form a rigid module, bind two distinct sites located on each subunit and *on the same face* of the dimer (Figure 3). Site I, on which bound MDGA-Ig1 covers a surface area of 859 A^2^, overlaps nearly completely with the neurexin-β binding interface. Site II, where MDGA-Ig2 covers a surface area of 1000 A^2^ (and which is split into two subsites in one of the studies), encompasses helices α2(7,8) and α3(7,8) in the four-helix bundle, along with the Cys-loop and L1-L2 loops which in neuroligin NLG4 are reorganized upon neurexin binding on the opposite face of the subunit [72], and which in AChE contribute to forming the PAS [38,62] (see above). Strikingly, the nature and spatial positioning of some ionic bonds at site I are conserved between bound MDGA1 and neurexin, a molecular mimicry reminiscent of that previously observed between Fab410 and Fas2 bound to the AChE PAS [40] (see above). Similarly, and although Fas2 does not bind neuroligins, structural overlay of the MDGA1-bound NLG with Fas2-bound AChE reveals that the tip of the βA-βB loop in MDGA1 Ig2 roughly coincides with the tip of loop II in Fas2. Incidentally, helix α3(7-8) that contributes to forming the MDGA-Ig2 binding site contains a laminin-binding Leu-Arg-Glu motif conserved in the dimerization domain of all neuroligins [27]. These data provide an unprecedented argument to why the neuroligins (and perhaps the ChEs, should a dual-binding ligand be identified in the future?) have to form dimers to be functional. Whether the other identified neuroligin partners, PTPRT, TSP-1, GluN1 (reviewed in [33]), punctin/MADD-4 [34,35], and Slitrk3 [36], interact directly with neuroligins, and if so, whether they share common binding sites and determinants with the neurexins and/or the MDGAs or instead associate with distinctive loci at the neuroligin surface, thereby extending further the partnership capability of the α/β-hydrolase fold domain, would be of interest.

### 2.5. The Back-Door Region

The “back door region”, which is remote from the gorge entrance and PAS at the AChE surface and would be distinct from the neurexin and MDGA binding surfaces on the neuroligin subunit, defines still another binding surface with functional relevance. Transient opening of a back door channel connecting the active center to the outside solvent, but distinct from the active center gorge, was proposed as a molecular mechanism contributing to the AChE high catalytic efficiency [81]. Concerted positional rearrangements of several residues in this region, including shutter-like motions of aromatic side chains forming a thin wall between the active center and the bulk, were then evidenced through combined structural and molecular dynamics simulation studies [82,83,84] (for a review see [17] in this issue). In turn, observation of open channels in the back door regions of crystalline DmAChE, aflatoxin-bound TcAChE and Fab410-bound BfAChE [40,85,86] and of substrate molecules bound in this surface region in a mAChE inactive mutant [87] led to support existence of a back door in AChE.

Monoclonal antibody Elec408, raised against EeAChE, was found to bind the EeAChE subunit specifically and non-competitively with PAS ligands Fas2, Elec403 and Elec410 and to inhibit it partially only (residual activity of the complex: ~30% of that of the unliganded enzyme), two features pointing to a new allosteric inhibition mechanism [63]. Mutational epitope mapping led to locate the Elec408 binding site near the putative back door [64] (Figure 2). In turn, the crystal structure of fragment Fab408, whose combining site encompasses a surface area of 1000 Å^2^ and is mostly populated by anionic side chains, led to further delineate the possible surface area and topography of the antibody binding site in the AChE back door region [65]. This region, which is distant by ~13 Å only from the shutter-like aromatic residues above mentioned, may provide a new target site for new specific modulators of AChE catalysis.

## 3. Conclusions

Would the α/β-hydrolase fold subunit herein exemplified by those of AChE, BChE, TG and neuroligin be approached as a cube, five of its six faces would correspond to a binding surface for a peptidic ligand: (i) the C-terminal face that accommodates the second molecule in the dimer; (ii) the PAS that binds toxin Fas2, antibodies Elec403/410 and MDGA-Ig2 (and perhaps the amyloid-β peptide and laminin); (iii) opposite to the PAS: the binding face for neurexin-LNS6 and MDGA-Ig1; (iv) between these two, on the same equatorial line: the back door region for Elec408 binding; (v) opposite to the back door region: the short Ω loop that mediates dimer-to-dimer contacts within the EeAChE loose tetramer and mAChE crystalline tetramer. The remaining sixth face, or N-terminal face, is not associated with a well-defined ligand, yet this is where the neurexin LNS4 domain might interact with the neuroligin surface to complement/reinforce primary LNS6 binding, where the ChE-like ectodomain of neurotactin is tethered to its transmembrane helical domain [88], and where one autism-linked substitution found in NL4 is located [27,89]. This is also where the ChE-like domain of TG is linked to the main body of the macromolecule, and where six TG substitutions associated with congenital hypothyroidism cluster ([48], and references therein). Hence, this sixth face of the α/β-hydrolase fold subunit may also play a functional role. An interesting feature of the AChE/BChE and ChE-like structures is the minimal interdigitation of the amino- and carboxyl-terminal domains, which facilitates formation of chimeras to investigate binding interfaces [12,90].

To conclude, this non-exhaustive review illustrates the interaction capacity and functional diversity conferred on the structurally conserved α/β-hydrolase fold core by those surface determinants that dictate partnership specificity for peptidic ligands. It also provides insights into existence of many interaction loci at the AChE subunit surface that might participate to its non-classical functions, thereby extending the range of possible target sites beyond the hitherto pinpointed PAS for the design of positive or negative effectors of these functions.

## Abbreviations

The following abbreviations are used in this manuscript:

AChE	acetylcholinesterase (BfAChE, from *Bungarus fasciatus* venom; EeAChE; from *Electrophorus electricus* electroplax; mAChE, recombinant from mouse; TcAChE, from *Torpedo californica* electroplax); BChE, butyrylcholinesterase (hBChE, recombinant from human)
CDR	complementary determining region
ChE	cholinesterase
EGF	epidermal growth factor
GPI	glycosyl-phosphatidylinositol
LNS	laminin-neurexin-sex hormone binding protein (domain)
MDGA	meprin, A5 protein, and receptor protein-tyrosine phosphatase mu [MAM] domain-containing GPI-anchored (protein)
NLG	neuroligin
PRAD	proline-rich attachment domain
PRiMA	proline-rich membrane-anchoring (domain)
WAT	tryptophan amphiphilic tetramerization (domain)
